# PutidaNET: Interactome database service and network analysis of *Pseudomonas putida *KT2440

**DOI:** 10.1186/1471-2164-10-S3-S18

**Published:** 2009-12-03

**Authors:** Seong-Jin Park, Jong-Soon Choi, Byoung-Chul Kim, Seong-Woong Jho, Jea-Woon Ryu, Daeui Park, Kyung-A Lee, Jong Bhak, Seung Il Kim

**Affiliations:** 1 Korean BioInformation Center (KOBIC), KRIBB, Daejeon 305-806, Korea; 2 Proteomics Research Team, Korea Basic Science Institute, Daejeon 305-333, Korea; 3 Graduate School of Analytical Science and Technology, Chungnam National University, Daejeon 305-764, Korea

## Abstract

**Background:**

*Pseudomonas putida *KT2440 (*P. putida *KT2440) is a highly versatile saprophytic soil bacterium. It is a certified bio-safety host for transferring foreign genes. Therefore, the bacterium is used as a model organism for genetic and physiological studies and for the development of biotechnological applications. In order to provide a more systematic application of the organism, we have constructed a protein-protein interaction (PPI) network analysis system of *P. putida *KT2440.

**Results:**

PutidaNET is a comprehensive interaction database and server of *P. putida *KT2440 which is generated from three protein-protein interaction (PPI) methods. We used PSIMAP (Protein Structural Interactome MAP), PEIMAP (Protein Experimental Interactome MAP), and Domain-domain interactions using iPfam. PutidaNET contains 3,254 proteins, and 82,019 possible interactions consisting of 61,011 (PSIMAP), 4,293 (PEIMAP), and 30,043 (iPfam) interaction pairs except for self interaction. Also, we performed a case study by integrating a protein interaction network and experimental 1-DE/MS-MS analysis data *P. putida*. We found that 1) major functional modules are involved in various metabolic pathways and ribosomes, and 2) existing PPI sub-networks that are specific to succinate or benzoate metabolism are not in the center as predicted.

**Conclusion:**

We introduce the PutidaNET which provides predicted interaction partners and functional analyses such as physicochemical properties, KEGG pathway assignment, and Gene Ontology mapping of *P. putida *KT2440 PutidaNET is freely available at http://sequenceome.kobic.kr/PutidaNET.

## Background

*P. putida *KT2440 is a ubiquitous bacterium which can break down a variety of organic materials for food. Because of its versatile metabolic activities, *P. putida *KT2440 is thought to play a pivotal role in the recycling of organic wastes and the degrading of biogenic and xenobiotic pollutants in the environment [1,2]. According to various carbon sources, we want to know the difference of networks according to the substrates. To simplify the culture condition, we selected succinate and benzoate as a sole carbon source. The easy carbon-utilization source, succinate and the required biochemical degradation-requiring benzoate were chosen for the comparison of a network analysis combined with the different proteomic data. An interactome of a species provides important clues about how to interpret metabolic pathways of constituent enzymes and global protein networks, which facilitates understanding the mechanism responsible for the cellular functions. Recently, the genomic-scale identification of protein-protein interaction (PPI) in model organisms, such as *Synechocystis *sp. PCC 6803 and *Xanthomonas oryzae*, have been published to map the whole protein-protein interaction networks [3,4]. Thanks to advanced high-throughput PPI experiments and information technology, many biologists can access large-scale species specific PPI data on the web [5]. Several web sites have been developed to disseminate PPI data such as POINT [6], OPHID [7], and PIANA [8]. POINT and OPHID systems provide predicted PPI information using sequence homology. PIANA integrates several proteins and interaction databases. However, these web services do not include the following methods: structure domain or domain-domain interaction, interaction networks in a graphical network viewer, functional annotation, localization, or the physicochemical properties of PPI data.

We constructed a web-based server, PutidaNET specifically for *P. putida *using major PPI algorithms. Functional and physicochemical annotations are provided using KEGG [9], Gene Ontology [10], amino acid distribution, instability index, isoelectric point, Gravy score, and sub-cellular localization. PutidaNET is designed to be user-friendly and easy to use.

## Methods

### Prediction of protein-protein interaction

The prediction of PPI is based on .PSIMAP [11,12], PEIMAP, and iPfam (domain-domain interaction) [4]. PSIMAP predicts interactions among proteins by using the BLASTP algorithm [13] with a common expectation value (E-value) cut-off of 0.0001. Interactions among domains or proteins for known PDB (Protein Data Bank) http://www.rcsb.org/pdb structures are the basis of the predictions. PEIMAP includes integrating various experimental protein-protein interaction databases such as BIND [14], DIP[15], IntAct [16], MINT [17], HPRD [18], CYGD) [19], and BioGrid [20]. PSIMAP and PEIMAP assume that, in terms of unknown proteins, the query tends to interact with its homolog's partners. The most commonly used concept is 'homologous interaction' [21-23]. In this step, we used to recruit homologous sequences using the PSI-BLAST [14] with a cut-off of 40% sequence identity. Furthermore, we have aligned the Pfam [24] domains of all the *P. putida *KT2440 proteins with hmmpfam by the cut-off of 0.01 (E-value).

In order to select more reliable PPIs, we developed and used a 'combined score' between any pair of proteins which were predicted by PEIMAP, PSIMAP, and iPfam algorithms. This scoring method is also used by the STRING server http://string.embl.de[25].

### Protein function annotation

In order to understand the biological function of *P. putida *KT2440 proteome, we searched physicochemical properties and cross-reference databases using KEGG and GO. We used Biopython [26] modules to acquire physicochemical properties, including hydropathy profile, GRAVY score (the average hydropathy score), molecular weight, amino acid distribution, isoelectric point, and protein instability index. In addition, we predicted trans-membrane helices and signal peptides using Phobius [27] and SignalP 3.0 [28] programs for the sub-cellular localization prediction of *P. putida *KT2440 proteome.

PutidaNET provides cross-reference to public database information such as 1) KEGG pathways, 2) GO categories, and 3) GO-slim [29] through protein ID mapping. In order to gain more accurate statistical test results of KEGG and GO assignment, we added Fisher's exact test algorithm (P-value).

### Protein network analysis case study

#### Cell culture and MS/MS analysis

In order to find significant features, we integrated PPI network and proteomic data which were produced as previously described [31]. *P. putida *KT2440 was pre-cultured at 30°C with vigorous shaking in culture media (50 mM potassium phosphate buffer, pH 6.25, 3.4 mM MgSO_4_, 0.3 mM FeSO_4_, 0.2 mM CaCO_3_, 10 mM NH_4_Cl, and 10 mM sodium succinate) and then inoculated into 1 L culture media containing succinate (10 mM) or benzoate (5 mM) as a sole carbon source. The bacteria were harvested at the late exponential phase (absorbance at 600 nm. 0.7-0.8) and suspended in 20 mM Tris-HCl buffer (pH 8.0). Bacteria were disrupted by a French pressure cell (SLM AMINCO, Urbana, IL) at 20,000 lb/in^2^, and soluble protein mixtures were prepared by centrifugation (15,000 g, 45 min). The protein samples were fractionated by 12% SDS-PAGE. The gel lanes were divided into 42 fractions according to molecular weight, and the sliced gels were digested with trypsin (Promega, Madison, WI). The resulting peptide extracts were pooled and lyophilized in a vacuum concentrator. Tryptic peptides were dissolved with 0.5% TFA (Trifluoroacetic acid) solution prior to further 2D-LC fractionation and used for MS/MS analysis using LTQ linear Ion Trap MS (ThermoFinigan San Jose, CA). For the database search, the *P. putida *protein database was downloaded from the National Center for Biotechnology Information http://www.ncbi.nlm.nih.gov/. Tryptic peptides were identified using SEQUEST (version 3.1 SR1, ThermoFinnigan).

For better accuracy of protein identification by MS/MS analysis, the *P. putida *protein database and the reverse protein database were used to exclude any false-discovered proteins [30].

#### sub-network

We acquired the protein lists in culture media including succinate or benzoate. In order to find regulated sub-network by succinate and benzoate, we analyzed betweenness centrality (BC), the number of shortest paths going through a certain node, and degree, the number of interaction partners, using NetworkAanalyzer, a cytoscape plugin [31]. We used the R software containing some packages and Welch two sample t-test for P-value [32]. Also, we found potential functional modules using MCODE, a cytoscape plugin that finds clusters (highly interconnected regions) in protein networks [33].

## Results

PutidaNET, a free accessible database with 3,254 proteins for *P. putida *KT2440, contains 82,019 PPI partners that have been predicted. Using the PPI algorithms, we obtained 61,011 (PSIMAP), 4,293 (PEIMAP), and 30,043 (iPfam) predicted PPIs except for self interaction. These PPIs were around 74.39% (PSIMAP), 5.23% (PEIMAP), and 36.62% (iPfam) of the *P. putida *KT2440 proteins. Although the total number of predicted interaction targets is very large, as they are ranked by combined score, experimentalists can select high ranking (more probable) ones according to their functional interests.

**Figure 1:**
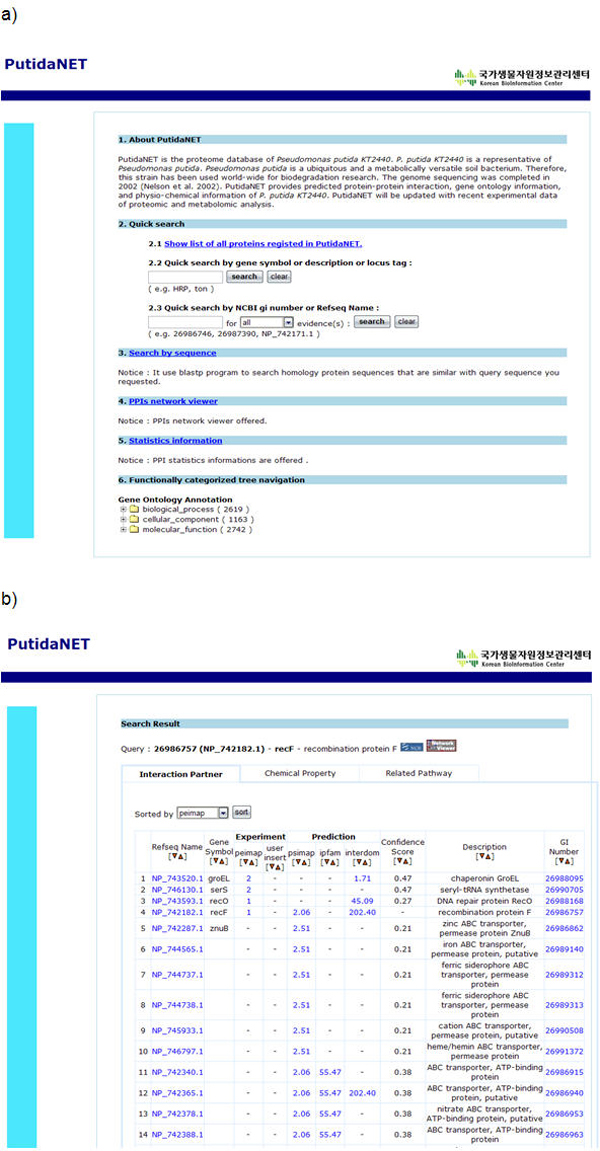
**PutidaNET system and interfaces**. (a) PutidaNET integrates three complementary protein-protein interaction databases including PSIMAP, PEIMAP, and iPfam. It shows three search interfaces: (1) search in high-confidence PPI network, (2) sequence search, (3) categorized tree navigation of gene ontology annotation. (b) A search result showing the list of predicted interacting proteins, confidence score, supporting description, and their synonymous IDs.

**Figure 2:**
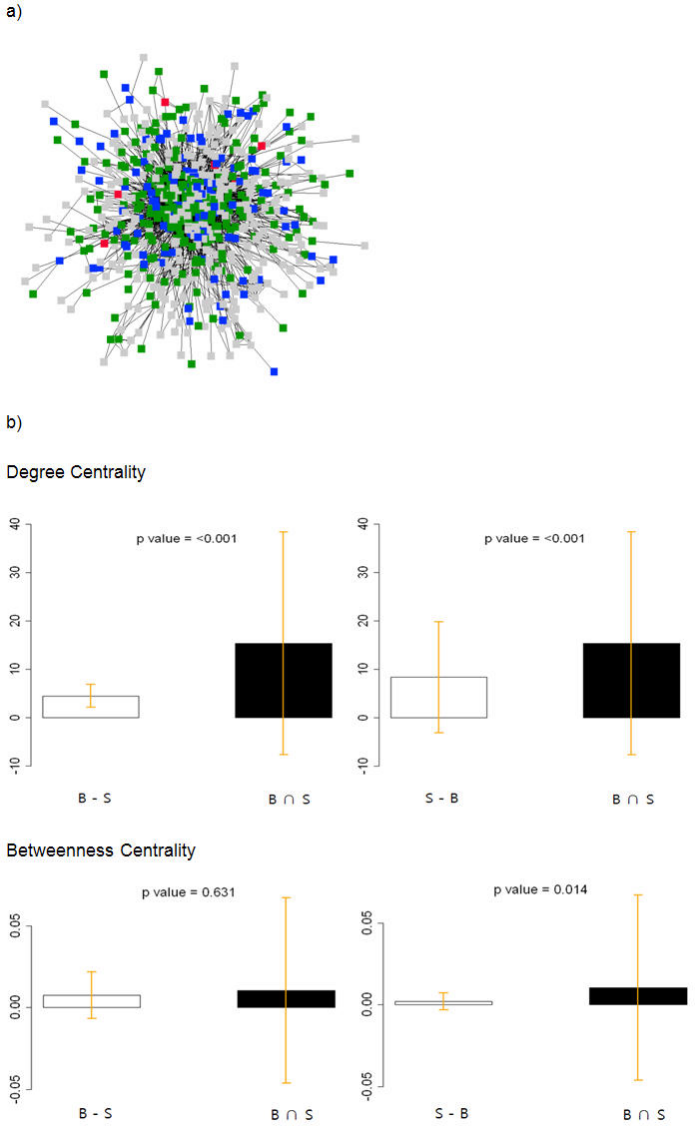
**Analysis of *P. putida *KT2440 protein interaction network**. (a) *P. putida *KT2440 protein interaction network from the proteomic data as previously described [1]. Red nodes represent the unique proteins when cultured in benzoate, except for certain proteins when cultured in succinate. Blue nodes represent the unique proteins when cultured in succinate, except for certain proteins when cultured in benzoate. Green nodes represent the commonly expressed proteins when cultured in either benzoate or succinate. Grey nodes have no information about Kim's mass information. (b) Average Scores of degree and betweenness centrality in green nodes are higher than those of only unique red and blue. This means that proteins when cultured in either benzoate or succinate regulate specific metabolism. We considered a P-value less than 0.05 to be statistically significant.

Figure 1 shows the search interface and the PutidaNET results. If a set of proteins is queried in the web interface, the user can acquire the physicochemical distribution against whole protein distribution, the trans-membrane protein abundances, and the queried protein set. Therefore, this summarized information can be used to evaluate the input data quality. The user can easily predict protein-protein interaction for queried proteins and examine protein-protein interactions with a network viewer made by JAVA. As a case study for PutidaNET, we used proteomics experimental data. As a measure of how central each protein is in the PPI network, we calculated two measures of betweenness centrality and degree for all the proteins in *P. putida *KT2440 [34]. And we colored the proteins which have mass abundance values in Figure 2a. From protein network analysis, we acquired some significant features about *P. putida *KT2440. PPIs were regulated specifically by difference sets of benzoate and succinate that tend to occur at the network periphery more than the network center (degree: *P*-value =< 0.001 and betweenness centrality: *P*-value = 0.014, Figure 2b). Also box plot indicates the each mean by categories. As well as error bar indicates the each confidence interval 95%. This implies that the main protein network of *Pseudomonas putida *KT2440 is regulated by an intersection set of succinate and benzoate. However, PPIs which were detected at the network periphery could be regarded as key regulation factors to use succinate or benzoate by *P. putida *KT2440. We expect that commonly induced proteins in succinate and benzoate media will be included in the essential metabolic pathways, which will be constitutively or continuously expressed regardless of culture conditions. Comparative analysis of 2-DE of *P. putida *KT2440 cultured in minimal medium (succinate) and rich medium (LB) also showed that the major induced protein patterns were very similar (data not shown). Specifically induced proteins in benzoate medium were β-ketoadipate pathway enzymes for benzoate and 4-hydroxybenzoate and stress proteins. On the other hand, enzymes for TCA cycle, pyruvate metabolism, and glycolysis were increased in succinate medium, which will be increased for the utilization of succinate influx.

**Figure 3:**
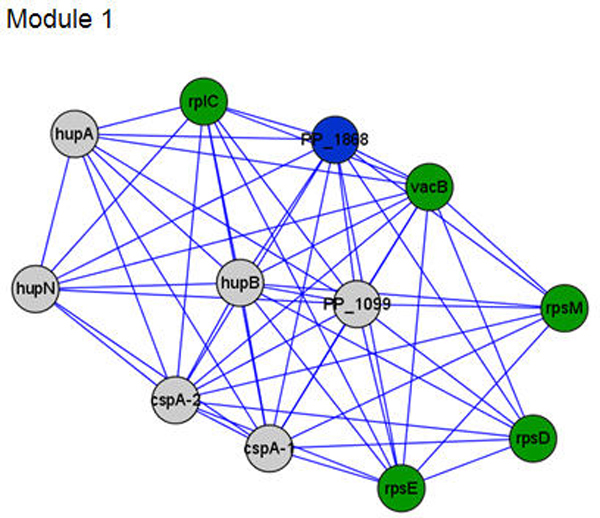
**The biological module obtained from MCODE cytoscape plug --in**. This figure shows functional module. For example, module 1 is a functional module about ribosome pathways. The node is a protein reference ID by NCBI. These modules compose cliques. We explained the meaning of node color in Figure 2 legend. The five functional modules show the figure in Additional File 1.

In order to find features in protein networks, we detected functional modules as highly interconnected sub-networks. As a result, we found five functional modules with KEGG pathway information (Figure 3, Additional file 1, Table 1). The functional modules are important PPIs because they represent protein complex or sub-pathway sharing biological functions. The modules which have less than a 0.001 P-value were various metabolic pathways and ribosomes. The metabolic pathway modules describe the characteristics of *P. putida *KT2440 which has a high level of metabolic diversity for biodegradation. This high level of diversity enables the bacterium to utilize a wide range of carbon sources. The ribosome is an organelle that coordinates protein synthesis in all cells. The bacterial ribosome consists of more than 50 ribosomal subunit proteins and three rRNAs. Since bacterial cells contain vast amounts of ribosomes, most ribosomal subunit proteins can be observed as main peaks by mass spectrometry.

**Table 1:** Pathway analysis of five modules obtained from MCODE

^a^**Module**	^b^**Pathway**	^c^**Matched proteins**	Total proteins	^d^**p-value**	^e^**Gene list**
**Module 1**	Ribosome	4	12	3.6E^-04^	rpsM, rpsD, rpsE, rplC

**Module2**	Ribosome	8	30	5.10E^-08^	rplV, rplA, rpsT, rplD, rpsC, rplM, rpsB, rpsA
	1,2-Dichloroethane degradation	3		5.90E^-04^	PP_2589, PP_3463, PP_2680
	Urea cycle and metabolism of amino groups	4		7.80E^-04^	PP_2589, PP_5278, PP_3463, PP_2680
	3-Chloroacrylic acid degradation	3		1.00E^-03^	PP_2589, PP_3463, PP_2680
	Ascorbate and aldarate metabolism	3		1.30E^-03^	PP_2589, PP_3463, PP_2680
	Glycerolipid metabolism	3		2.90E^-03^	PP_2589, PP_3463, PP_2680
	Butanoate metabolism	4		3.00E^-03^	PP_2589, PP_3463, PP_2680, ilvB
	Bile acid biosynthesis	3		3.70E^-03^	PP_2589, PP_3463, PP_2680
	Histidine metabolism	3		6.70E^-03^	PP_2589, PP_3463, PP_2680
	Limonene and pinene degradation	3		7.30E^-03^	PP_2589, PP_3463, PP_2680
	beta-Alanine metabolism	3		8.60E^-03^	PP_2589, PP_3463, PP_2680

**Module3**	ABC transporters - General	4	9	3.8E^-03^	PP_0225, aapP, PP_1068, PP_5022

**Module4**	Ribosome	8	45	9.9E^-07^	rplI, rplB, rplQ, rplL, rpmB, rplP, rplE, rplX
	Glycolysis/Gluconeogenesis	4		4.1E^-03^	aceE, eno, aceF, lpdG

**Module 5**	Ribosome	6	30	2.8E^-05^	rpsG, rpsN, rplR, rplS, rplW, rpsH

^a^We made five modules using MCODE.

^b^We analyzed pathway analysis using KEGG pathway information.

^c^Count column displays the number of protein containing each pathway in module.

^d^The p-value is confidence score by Fisher's exact test algorithm.

^e^Gene list column shows gene symbols containing each pathway.

## Conclusion

PutidaNET is the integration of mutually complementary protein-protein interaction information for the systematic analysis of *Pseudomonas putida*. The PutidaNET server is the first web server that provides various kinds of functional information such as a PPI viewer, physicochemical properties, biological pathways, gene ontology, and protein-protein interaction for *P. putida *KT2440. It can assist researchers to access and obtain the information through an automatic annotation for queried proteins. Using proteomics data from certain medium conditions, we analyzed the characteristics of *P. putida *KT24400 using PutidaNET. Proteomic data gave us the quantitative information of induced proteins at benzoate or succinate culture conditions, which supplements the database. PPI combined with proteomic data can give users more specific information.

## Competing interests

The authors declare that they have no competing interests.

## Authors' contributions

SJP, BCK, and JWR constructed the database. SWC and DP developed the web site. JSC designed the overall web site. KAL analyzed the statistics of PPI network results. SJP, JSC, DP, and JWR wrote the main draft of the paper. JB and SIK directed the study and helped with the draft manuscript.

## Note

Other papers from the meeting have been published as part of *BMC Bioinformatics *Volume 10 Supplement 15, 2009: Eighth International Conference on Bioinformatics (InCoB2009): Bioinformatics, available online at http://www.biomedcentral.com/1471-2105/10?issue=S15.

## Supplementary Material

Additional file 1 **Supplementary Figure 1-(a-e) the biological modules obtained from MCODE cytoscape plug - in**. This figure shows five functional modules: a) module 1, b) module 2, c) module 3, d) module 4, and e) module 5. For example, module 1 is a functional module about ribosome pathways. Click here for file
