# Supplementary material for: PutidaNET: Interactome database service and network analysis of Pseudomonas putida KT2440
Source: BMC Genomics. 2009 Dec 3;10(Suppl 3):S18. doi: 10.1186/1471-2164-10-S3-S18 (PMC2788370; doi:10.1186/1471-2164-10-S3-S18)

SUPPLEMENTARY INFORMATION

**Figure 1-(a-e) the biological modules obtained from MCODE cytoscape plug - in**

This figure shows five functional modules: a) module 1, b) module 2, c) module 3, d) module 4, and e) module 5. For example, module 1 is a functional module about ribosome pathways.

**a) Module 1**


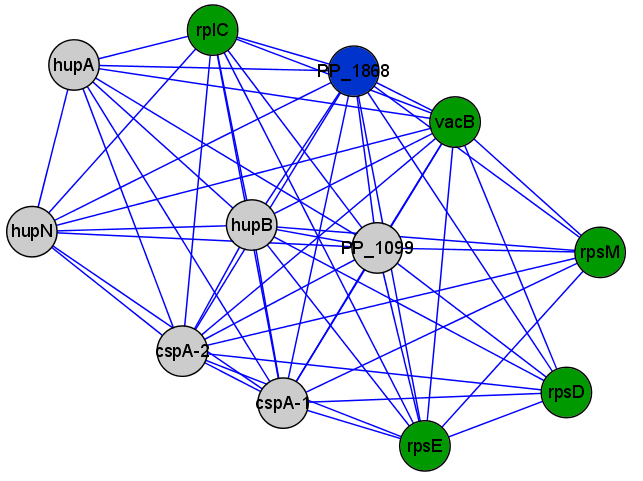


**b) Module 2**


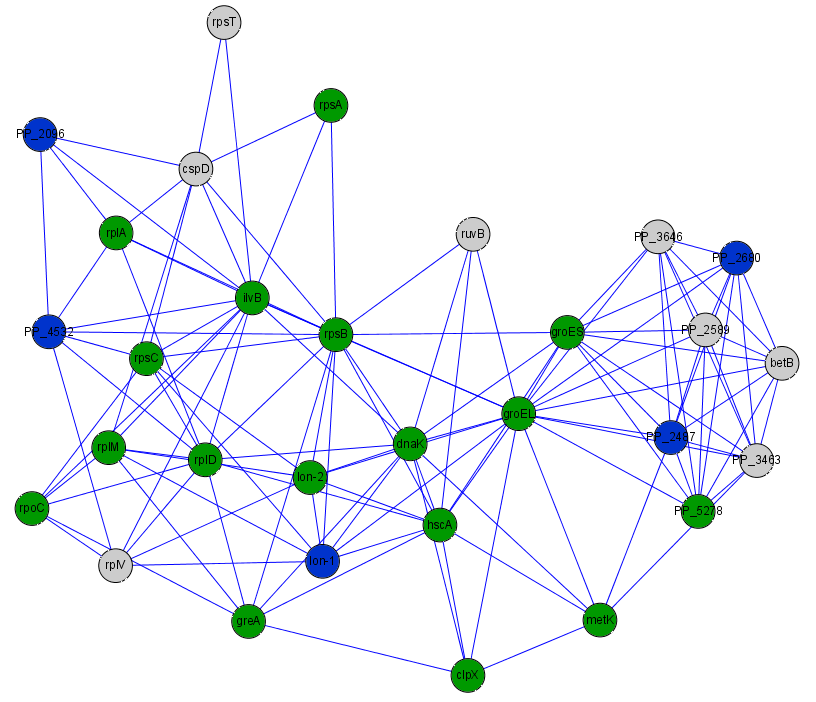


**c) Module 3**


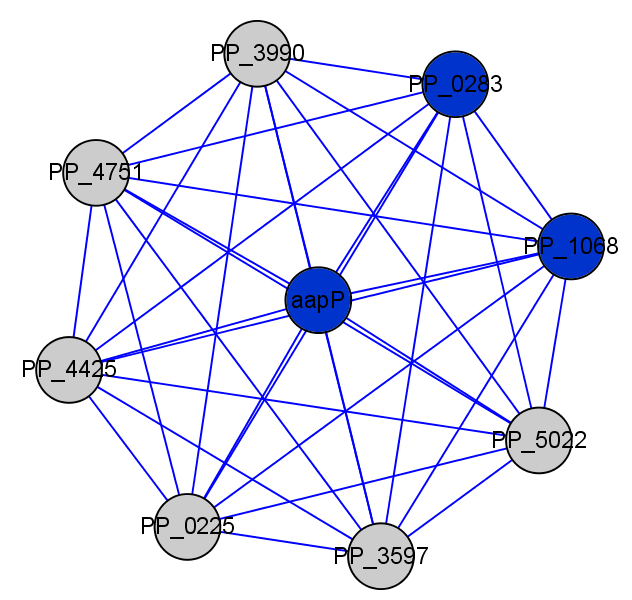


**d) Module 4**


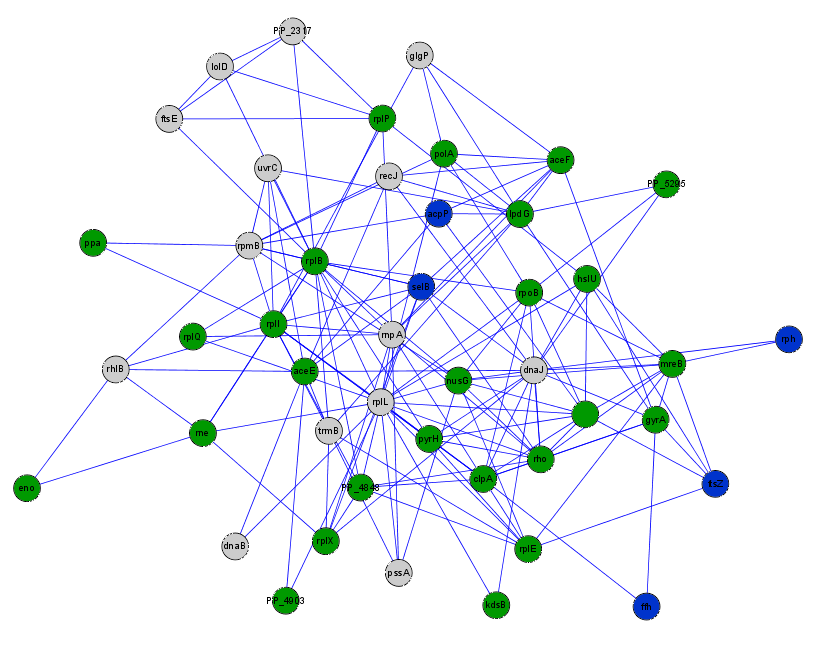


**e) Module 5**


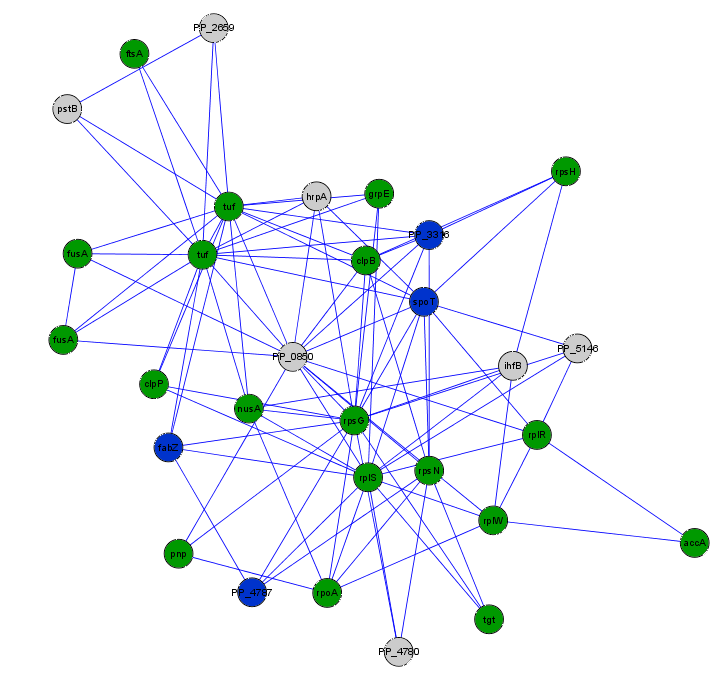

Supplement: Additional file 1 — Supplementary Figure 1-(a-e) the biological modules obtained from MCODE cytoscape plug - in. This figure shows five functional modules: a) module 1, b) module 2, c) module 3, d) module 4, and e) module 5. For example, module 1 is a functional module about ribosome pathways. [file 1471-2164-10-S3-S18-S1.doc]
